# Combining Ground Based Remote Sensing Tools for Rockfalls Assessment and Monitoring: The Poggio Baldi Landslide Natural Laboratory

**DOI:** 10.3390/s21082632

**Published:** 2021-04-08

**Authors:** Saverio Romeo, Antonio Cosentino, Francesco Giani, Giandomenico Mastrantoni, Paolo Mazzanti

**Affiliations:** 1 Department of Earth Sciences, University of Rome “Sapienza”, Piazzale Aldo Moro 5, 00185 Rome, Italy; saverio.romeo@uniroma1.it (S.R.); cosentino.1666074@studenti.uniroma1.it (A.C.); giani.1658368@studenti.uniroma1.it (F.G.); paolo.mazzanti@uniroma1.it (P.M.); 2 NHAZCA S.r.l., Via Vittorio Bachelet 12, 00185 Rome, Italy

**Keywords:** landslide monitoring, TInSAR, gigapixel, acoustic signal, remote sensors, remote sensing

## Abstract

Nowadays the use of remote monitoring sensors is a standard practice in landslide characterization and monitoring. In the last decades, technologies such as LiDAR, terrestrial and satellite SAR interferometry (InSAR) and photogrammetry demonstrated a great potential for rock slope assessment while limited studies and applications are still available for ArcSAR Interferometry, Gigapixel imaging and Acoustic sensing. Taking advantage of the facilities located at the Poggio Baldi Landslide Natural Laboratory, an intensive monitoring campaign was carried out on May 2019 using simultaneously the HYDRA-G ArcSAR for radar monitoring, the Gigapan robotic system equipped with a DSLR camera for photo-monitoring purposes and the DUO Smart Noise Monitor for acoustic measurements. The aim of this study was to evaluate the potential of each monitoring sensor and to investigate the ongoing gravitational processes at the Poggio Baldi landslide. Analysis of multi-temporal Gigapixel-images revealed the occurrence of 84 failures of various sizes between 14–17 May 2019. This allowed us to understand the short-term evolution of the rock cliff that is characterized by several impulsive rockfall events and continuous debris production. Radar displacement maps revealed a constant movement of the debris talus at the toe of the main rock scarp, while acoustic records proved the capability of this technique to identify rockfall events as well as their spectral content in a narrow range of frequencies between 200 Hz to 1000 Hz. This work demonstrates the great potential of the combined use of a variety of remote sensors to achieve high spatial and temporal resolution data in the field of landslide characterization and monitoring.

## 1. Introduction

Varnes in 1978 described the rockfall process as “a detached fragment of rock that falls along a vertical or sub-vertical cliff, proceeds down slope by bouncing and flying along ballistic trajectories or by rolling on talus or debris slopes” [1]. Rockfalls are the most frequent and widespread instabilities affecting steep slopes in mountain regions and sea cliffs [2,3,4,5,6]. Therefore their impact energy and the associated hazard can reach very high values [7,8,9,10,11,12,13]. According to [14], along the Italian mountain ranges (Alps and Apennines), fast moving landslides such as rockfalls caused the largest number of deaths. As demonstrated in recent studies [15,16,17,18,19,20,21,22,23], surveying and monitoring activities play a key role in rockfall hazard assessment and are therefore of paramount importance for the preservation of human life. As rockfalls occur in very steep slopes sometimes showing limited precursor signals [24,25,26,27,28,29], their investigations, as well as their prediction and stabilisation, could face many difficulties [30]. Conventional geotechnical instrumentations like extensometers, inclinometers and piezo-electric transducers recording acoustic emissions (AE) caused by rock fracturing are currently used in rock slope monitoring with positive results [31,32,33,34,35,36,37]. In detail, acoustic emissions have the potential to identify ongoing deformations affecting the slope, representing a key component in the development of early-warning systems [33,38]. AE is generated during slope movements and acoustic monitoring is often capable of detecting pre-failure deformations [39]. However, the installation of contact sensors on steep and unstable slopes may be expensive and hazardous. Hence, rockfall assessment and monitoring can be carried out using remote sensing techniques. Techniques like Terrestrial Laser Scanning (TLS) and Aerial Photogrammetry [4,40,41,42,43] are becoming more and more used for volumetric analyses of rock falls [44,45,46,47], geomechanical characterization [21,48,49,50,51] and long-term monitoring of slow movements [52,53]. However, such technologies suffer some limitations for real time monitoring implementation (e.g., automated data processing, measurement repeatability, reliability, etc.). Therefore, their implementation in early warning systems has not been fully achieved [54,55].

In this context, this work shows the field activities performed at the Poggio Baldi Landslide Natural Laboratory (hereafter referred as PBL) with the aim to combine data acquired from different ground-based remote sensing sensors for rockfalls assessment and monitoring. The PBL, developed since 2015 by the joint effort of the Department of Earth Sciences of “Sapienza” University of Rome” and NHAZCA Srl [56], is today an ideal environment for testing the potential of different remote sensing technologies thanks to the frequent rockfall activity of its scarp.

The paper discusses the results of a three-day intensive monitoring campaign (14–17 May 2019) based on continuous Terrestrial Interferometric SAR (TInSAR) data (i.e., every 30 s for 72 h), multi-temporal Gigapixel images and acoustic records. Real-time analysis was carried out for TInSAR data, while optical Gigapixel images and acoustics data were subsequently processed by means of change detection and frequency analysis respectively. These monitoring activities were performed at PBL with the purpose of assessing the potential gains from the combined use of ground-based remote sensors and improving the knowledge of the ongoing gravitational processes.

In addition to the Introduction Section, the paper is organized in five main chapters. The Materials and Methods Section describes the employed methodology and equipment used. In Section 3 the geological and geo-morphological setting of PBL is described as well as its recent rockfall activity. Section 4 shows the main results of this field-work and the results are analysed in Section 5. Finally, the main conclusions are presented in Section 6.

## 2. Materials and Methods

**Figure 1:**
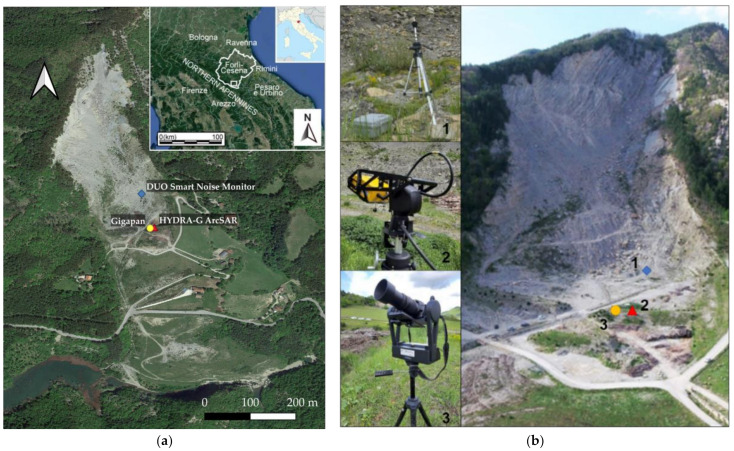
Operational ground-based remote sensing tools during the intensive monitoring campaign at Poggio Baldi Landslide Natural Laboratory (PBL): (**a**) Top view of the slope including the location of monitoring instruments. Upper right inset with enclose the geographical frame of the Forlì-Cesena province in the Northern Apennines; (**b**) UAV picture of the Poggio Baldi landslide upper slope and the corresponding positions of 01dB DUO Smart Noise Monitor (1), HYDRA-G ArcSAR (2) and GigaPan Epic Pro V system (3) about the rock cliff under study.

In this section, the monitoring techniques used during the three-day monitoring campaign at PBL are presented including their technical specifications, settings and location on-field (Figure 1). It is worth highlighting that innovative tools, at the disposal of the research team, were used for the first time in an integrated way for an intensive monitoring campaign.

Regarding the methods used, before the field activities (i.e., data acquisition stage) a reasonable amount of time was dedicated to the designing of monitoring activities to be carried out, taking into account the specific conditions of the landslide process, previous studies and evidence from past field surveys. In detail, the definition of the main parameters to be monitored, the identification of the most appropriate technological solutions and the setting of measurement procedures with their on-field operational activities were carried out.

In this way, a systematic approach to planning the monitoring network was applied [57]. The planning activity started by analyzing and defining the project conditions which included the analysis of existing documents, data and literature describing the recent history of the landslide process. Focus was on physical parameters such as those which were objects of the intensive monitoring campaign: surface movements, sounds produced by cracks/joints opening and rockfalls. All these conditions were integrated with available geological and geotechnical information to provide for a comprehensive picture of the situation to deal with. In addition, given the remote location of the landslide, particular focus was paid to logistics aspects (e.g., power supply, telecommunications, safety of workers, etc.).

### 2.1. GigaPan

**Figure 2:**
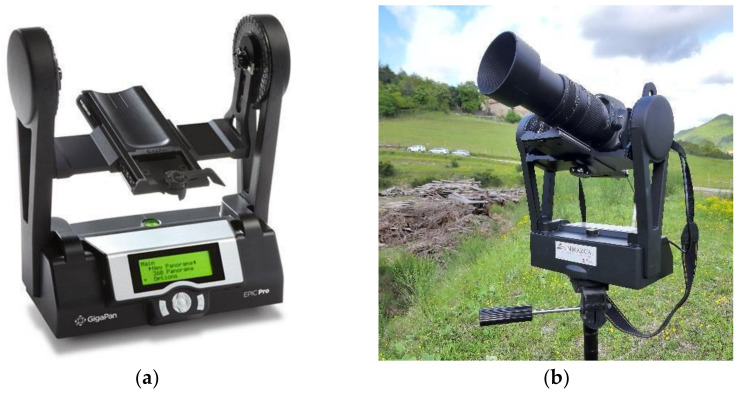
GigaPan acquisition system used at PBL: (**a**) GigaPan robotic head with screen and controls; (**b**) GigaPan system equipped with a Nikon D5000 camera and 300mm telephoto lens.

Gigapixel identifies a digital image achieved by stitching technique, i.e., by merging several images acquired from the same position covering the whole view of interest. These digital images can be composed of billions of pixels or more (equal to or greater than 1000 Megapixels) [58,59,60]. As commercial DSLR cameras generally use sensors with more than 20 million pixels (20 Megapixels), to create a Gigapixel image it is required the use of tool like GigaPan EPIC PRO, produced by GigaPan Systems LLC [61]. GigaPan was created in 2008 by a research collaboration between NASA and Carnegie Mellon University to develop a high-resolution imaging technique for use in the Mars Exploration Rover mission. In short, the GigaPan system allows the sequential acquisition, using a robotic head (Figure 2), of hundreds of partially overlapped (40–80%) images, thus avoiding parallax errors. This result is achieved thanks to a robotic head mounted on a tripod that allows the rotation of the camera around the point of no parallax (NPP) [62,63]. The overlapping areas are exploited by the stitching software to determine the points of correspondence, from which starts the reconstruction of the image and the rearrangement of the pixels’ number. To date, this technique saw its greatest use in geosciences as a qualitative way for the representation and interpretation of high-resolution scenarios [64]. The use of Gigapixel images as raw data processed by innovative PhotoMonitoring techniques (e.g., using Change Detection algorithms) may represent an effective starting point for quantitative analysis for assessing and monitoring purposes. That means capturing high-resolution images over time from a fixed position and comparing them, thus performing a 2D Change Detection (2D-CD) [65,66,67].

For the present case study, the high-resolution images in RGB colors have been acquired by the GigaPan Epic Pro V system [68] (produced by GigaPan Systems LLC), equipped with:

- Digital camera Nikon D5000 with 23.6 × 15.8 mm CMOS sensor (12.3 Megapixel).
- Telephoto lens with variable focal length up to 300 mm.

The single shots, acquired up to a maximum distance of 700 m from the cliff, were processed through the stitching software Image Composite Editor (ICE, Microsoft) [69]. The stitched images were represented by an original resolution of 2.2 gigapixel. However, in order to facilitate further processing operations, the images have been resampled to a lower resolution (572.28 megapixels) and finally exported. Once the stitching phase was completed, the images were imported into the free software QGIS to carry out the 2D-CD through the co-registration made by the georeferencing and the raster alignment tools. Within the 2D-CD, rockfall areas were detected and measured, and finally enclosed into a unique vector file. For such analysis, the GigaPan system was used from the same position with a time interval of two hours during the monitoring campaign (daytime only).

### 2.2. Terrestrial ArcSAR Interferometry

In the last decades, one of the most effective technologies among landslide monitoring systems has been Terrestrial SAR Interferometry, which exploits the interferometric technique (i.e., by comparing the phase signal difference between two or more images collected at different times) measuring the displacement along the Line of Sight (LOS). This technology has several advantages, including the capability to collect data under any weather and lighting conditions, a high data sampling rate (up to few seconds), and does not require direct interaction with the investigated area (e.g., targets are not mandatory) [70,71,72,73]. The first applications of SAR interferometry (InSAR) appeared in the early 1990s, using radar images taken from satellites to measure displacements on the ground [74,75,76,77,78,79]. Terrestrial SAR interferometry (TInSAR) also referred as Ground-Based Interferometric Synthetic Aperture Radar (GB-InSAR), is a technique for displacement monitoring based on the same principles of satellite-based InSAR [78,79,80,81,82,83,84]. TInSAR is an active microwave acquisition system operating in the Ku-band (12–18 GHz) which uses phase measurements of the radar signal to detect displacements of targets; therefore, it is able to acquire images in any weather conditions during both day and night, and for each pixel of a SAR image, a phase value is measured along the LOS of the radar [85,86,87,88,89].

Differently from conventional SAR systems for landslide monitoring, consisting of radar antennas that move along a linear scanner, Terrestrial ArcSAR Interferometry uses sensor motion along a circular trajectory (e.g., along an arc) to create the synthetic aperture [88,90]. The ArcSAR configuration (i.e., radar antenna placed on a tripod which follows a circular movement) has solved many drawbacks of conventional TInSAR, such as large sizes, heavy weights, and significant on site infrastructure requirements [91]. Basically, ArcSAR allows to retrieve better resolution with smaller antenna [92,93].

**Figure 3:**
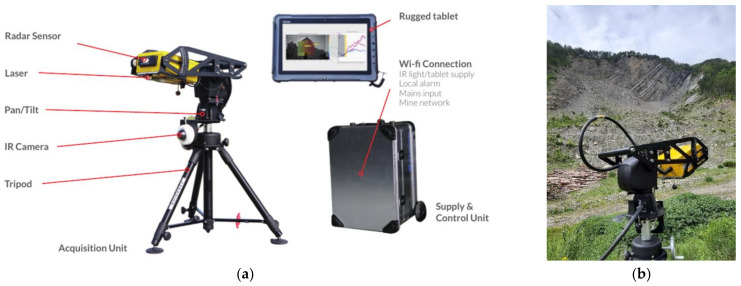
(**a**) HYDRA-G equipment; (**b**) Terrestrial ArcsSAR Interferometry installed at PBL.

In the framework of the present study, a compact ArcSAR called HYDRA-G designed and manufactured by IDS GeoRadar (part of Hexagon) was used (Figure 3). Such an instrument is able to provide displacement measures with a sub-millimetre accuracy, by combining a very high phase measurement accuracy (<0.1 radians) and a short wavelength (4 mm) [55]. Its maximum range is about 800 m with a field of view up to 120° (horizontal) and 30° (vertical) and its operating temperature is −20 °C to +55 °C. The image resolution is 0.2 m in range and 14 mrad in cross-range. The maximum sampling interval is of about 30 s. The instrument is also equipped with an optical and infrared HD camera and with a short-range laser scanner in order to provide real-time images and to autonomously reconstruct the geometry of the monitored scenario.

The equipment was deployed within the monitoring site of PBL in the vicinity of the other instruments used. The selection of the radar location represented a crucial factor for the monitoring activities and data interpretation as the radar measurements are computed only along the LOS. In detail, the radar with its tripod was placed in a stable and safe area about 400 m away from the scarp in order to obtain a field of view including both the vertical rock cliff and the debris talus at its toe (Figure 1). The radar measurements started on 14 May 2019 from 11:00 (UTC+ 01:00) and ended on 17 May 2019 at 11:15 (UTC+ 01:00) for a total of 72 h of monitoring.

### 2.3. Acoustic Measurements

**Figure 4:**
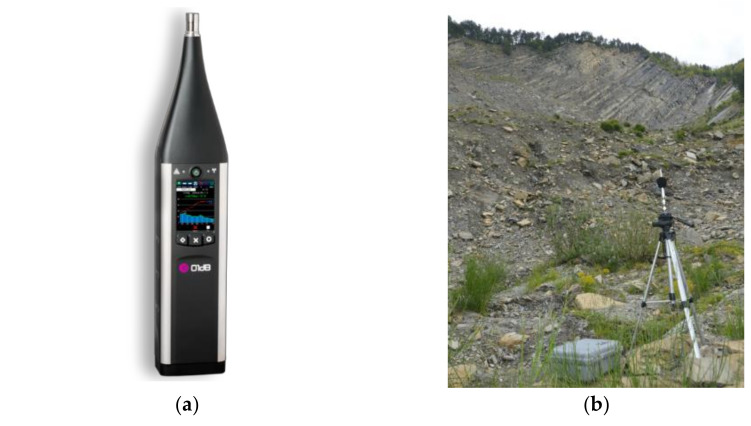
Overview of the 01dB DUO Smart Noise Monitor: (**a**) DUO instrument body in LOG mode; (**b**) Outdoor microphone unit DMK01 and the waterproof case DSC01 containing the instrument body during the rockfall monitoring campaign carried out at the PBL.

The acoustic signal measurements were carried out on the sound level meter DUO Smart Noise Monitor (FW 2.45, class 1 IEC-61672 certification) produced by 01dB company (part of ACOEM Group, Limonest, Rhône, France) and combined with an outdoor microphone unit of type DMK01, especially designed to split the microphone from the instrument body. This unit is made of a stainless-steel body, a dedicated preamplifier (PRE22) connected to the external output, a noise cone and a specific windscreen (Figure 4). The 01dB DUO can measure one-third octave band noise levels down to 6.3 Hz together with recording audio samples up to 180 dB at set intervals.

Regarding acoustic landslide monitoring, instantaneous sound pressure levels (dB) and spectra (Hz) have been recorded [94,95]. In this way we could analyse and classify them by using the proprietary software dBTrait 6 in post-processing LOG mode (Integrating Logging sound level meter), a kind of signal capture which includes the storage of time histories.

According to the aim of identifying rockfall occurrence and its spectral signatures, we recorded both sound pressure levels and frequencies on 15 May 2019 from 18:00 to 19:00 (UTC+ 01:00) and on 16 May from 12:40 to 15:10 (UTC +01:00), installing the microphone unit on the lower part of the debris generated by the rock cliff (Figure 4b). These activities were allowed thanks to the waterproof DSC01 case which provide complete protection to the DUO instrument also for mid- and long-term environmental sound measurements. With this configuration, the external microphone DMK01 with its tripod was the only outdoor instrument.

Once rockfall events were recognized and classified using the dBTrait software, we were able to visualize their spectra with frequency-weightings of type “Lin”, and compare them with each other and with environmental sounds like birds, water runoff and aircraft noise, with the aim of defining the spectral contents of rockfalls arising from the Poggio Baldi rock cliff. Audio tracks of rockfall events and other environmental noises are available as supplementary materials.

## 3. Test Site of Poggio Baldi Landslide

### 3.1. General Framework

The PBL is located in the municipality of Santa Sofia (northern Apennines, Emilia Romagna Region, Italy), close to the Corniolo village, and it is reported in Sheet no. 265 “S. Piero in Bagno “scale 1:50000 of the geological map of Italy [96]. The Poggio Baldi landslide develops from the slopes of the homonymous mountain (43.910538° N, 11.807625° E), on the hydrographic left of the Bidente river, to the stream below, and it represents one the most prominent geomorphological features of the Bidente Valley. The first activation dates to 25 March 1914, whereas the last reactivation of the landslide deposit took place on 18 March 2010, and it was triggered due to the increase of water pressure in the pores of the paleo-landslide’s body following the melting of the snow blanket caused by a sudden increase in temperatures. The landslide has an estimated volume of about 4 × 10^6^ m^3^ [56], and it is currently active in its upper scarp due to frequent rockfall events [47].

Thanks to its distinctive features, which are representative of many landslides worldwide in terms of material involved, type of movement and triggering factors, the PBL was established in 2015 [56] with the aim of testing innovative remote sensing technologies in such a characteristic environment [47,97].

### 3.2. Geological and Geomorphological Setting

**Figure 5:**
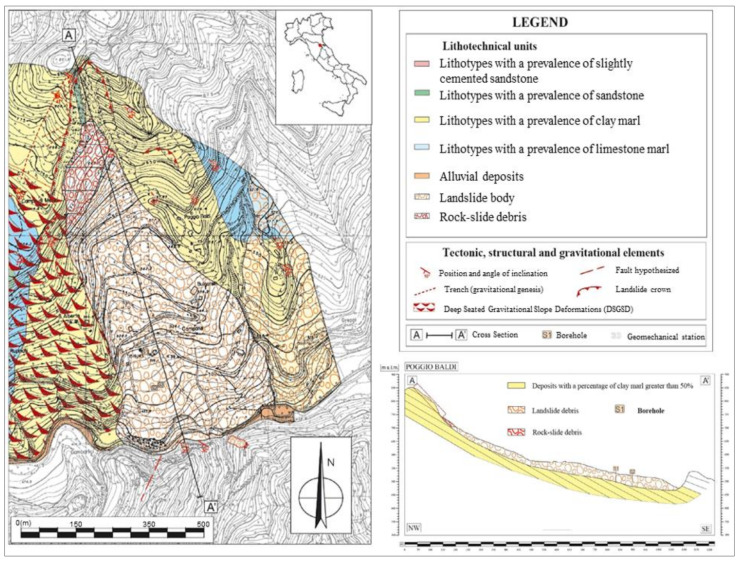
Geological map of the Poggio Baldi landslide area with a related cross-section along its body. The investigated rockfalls arise from the rock cliff outlined by the red dashed line and by the red box in the cross-section; modified from [47].

The slope involved in the Poggio Baldi landslide is a part of the hanging-wall of a major thrust system (i.e., San Benedetto in Alpe) and is composed by the Marnoso-Arenacea Formation (Miocene), involving an alternation of claystone, siltstone and sandstone, arranged in a monoclinal dip slope sequence (Figure 5) [56,97,98,99,100,101]. Landslides involving Flysch sequences, which are characterized by geo-lithological complexity and heterogeneity, are widely diffused in the northern Apennines as well as in other mountain chains [102,103,104,105]. A slight bending of the strata occurs in the lower part of the slope: the bedding attitude, dipping at about 45° upslope, progressively decreases reaching dip angles of about 15–20° downstream. A set of high angle, normal faults oriented roughly perpendicular to the main thrust geared NW-SE complete the structural frame [56,97,99,106]. The geomorphology of this sector is strongly controlled by the structural setting and the recent tectonic activity. Thanks to the progressive erosion of the Bidente river, gravitational processes, featured by different landslide types with a very wide range of dimensions, are one of the most significant morpho-genetic factors of the area.

According to [47,56,97], the landslide can be classified as a complex process initially set as a rock wedge slide (1914) followed by rockfall processes arising from the resulting head scarp. Following a long period (about a century) of rock debris accumulation on top of the landslide deposit, a critical overload, combined with intense and prolonged rainfall and snow melting, led to reactivation of the landslide deposit (2010) which evolved into a composite movement presenting rotational debris, earth slide [1,107] and flow slide-like movement [56,97,108,109]. The landslide was also favoured by the structure dip out of the outcropping rocks. The main rock slope, the target of this study, is a sub-vertical scarp with a rise of about 100 m and a width of 250 m and it is characterized by high-frequency rockfall processes.

### 3.3. The Occurrence of Recent Rockfall Events

**Figure 6:**
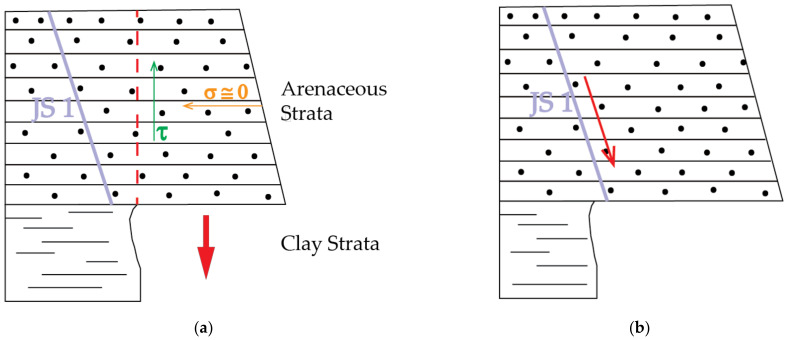
Conceptual model related to the two main triggering mechanisms of rockfall phenomena on the Poggio Baldi rock cliff: (**a**) Breaking of the arenaceous mass due to shear strength (τ) exceedance; (**b**) Failures due to planar sliding along the JS 1 discontinuity set.

Since the first activation in 1914, despite the following steady state of the main landslide body, the newly formed vertical rock cliff has always been frequently affected by rockfalls, which accumulate at the base of the scarp generating a debris talus which continuously increases its volume [47]. The instability is predisposed by geologic, geomorphologic and structural factors, such as the alternance of arenaceous and clayey strata and the presence of several discontinuities. In [97] it is demonstrated that the bedding is the main discontinuity system on the slope face and the joint set N108/73° (here called JS 1), is the prevalent joint system. Using the Markland method [110] different failure mechanisms like translational sliding movements of rock wedges, translational planar sliding and direct toppling were detected. Moreover, considering the progressive erosion of the clay strata, which gradually increases the overhang of the arenaceous strata, rockfalls are mainly triggered by: (i) the breaking of the arenaceous mass due to shear strength exceedance (Figure 6a) and, (ii) the intersection of a JS 1 joint with the exposed surface of the cliff (Figure 6b). The detached debris and blocks often find deposition surfaces over the underlying arenaceous strata. Over time, the volume of fallen material increases on the overhanging strata till the debris friction angle (φ = 15°) is exceeded, with the consequent remobilisation, or the strata collapsing. These conditioning factors, combined with each other, are the main cause of the widespread instability throughout the vertical rock cliff. As a matter of fact, [47] estimates the general loss of volume from the vertical rock cliff to be in the range of 2.0 to 2.8 × 10^3^ m^3^ per year.

## 4. Results

### 4.1. GigaPan

**Figure 7:**
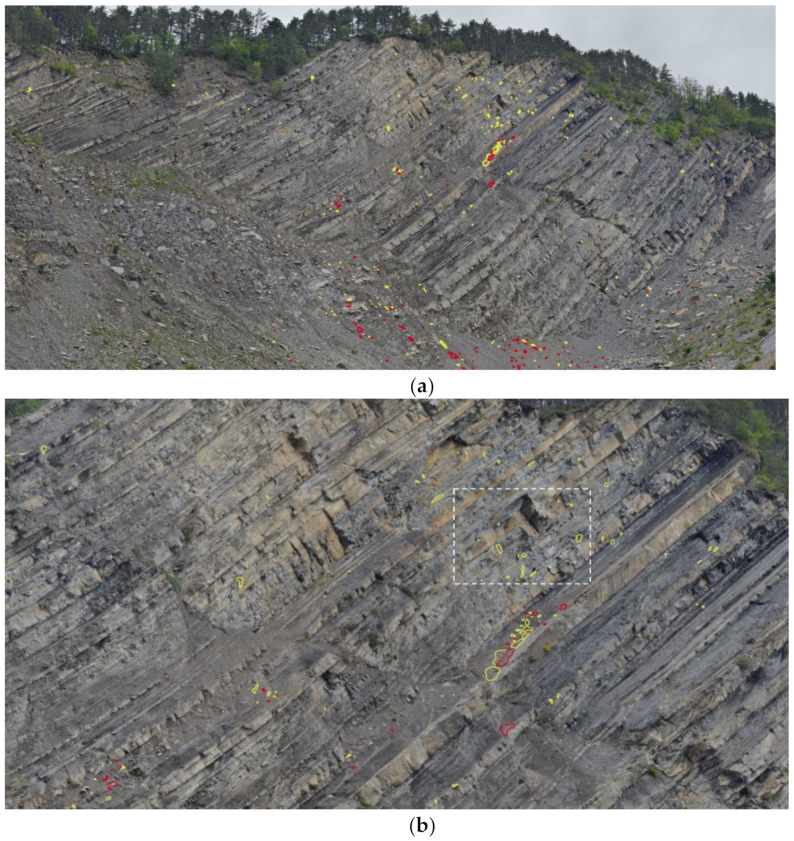
Gigapixel image with all rockfalls detected during the three days monitoring campaign: (**a**) 2D Change Detection (2D-CD) along the Poggio Baldi cliff with detached rock falls (yellow) and debris removal (red); (**b**) Zoom of the most active sector during the monitoring campaign.

The 2D-CD from GigaPan images allowed us to identify and map a total number of 84 rockfalls, developed in the three days of monitoring, with 32 rockfalls occurring on the first day, 9 rockfalls on the second day and 43 on the third day of monitoring. The most active portion of the rock cliff between 14 and 17 May 2019 was the central one, and it is pointed out in Figure 7.

**Figure 8:**
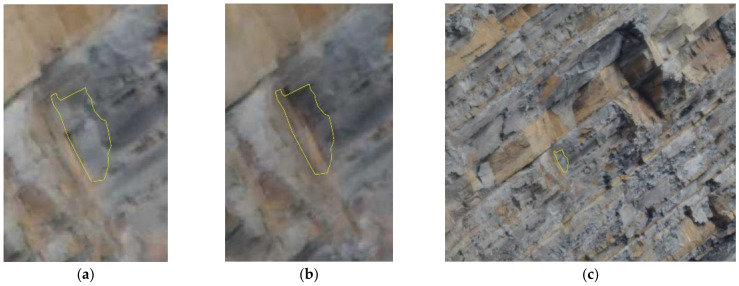
Example of a rockfall which occurred on 16 May 2019 between 11:50 and 13:50 local time: (**a**) Image of 11:50; (**b**) Image of 13:50; (**c**) Location of the rockfall event in the cliff (white-dashed box in Figure 7b).

Using the different Gigapixel images, rockfalls were dated with an accuracy of two hours. For example, the rockfall depicted in Figure 8, having a surface of about 0.5 m^2^, occurred on 16 May 2019 between 11:50 and 13:50. To calculate the surface of the rockfall and its size, it was necessary to define the Ground Pixel Size (GPS) using the 3DM CalibCam software and an object distance calculation spreadsheet tool, knowing the exact distance between the GigaPan station and the rockfall source zone.

**Figure 9:**
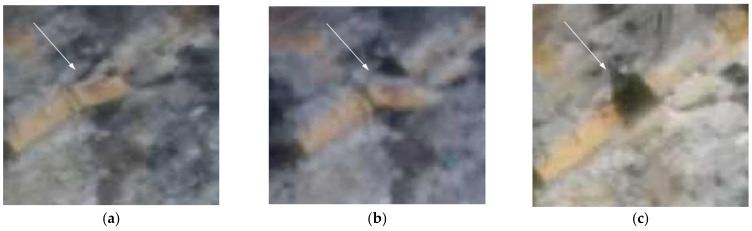
Evidence of precursory movements before the rockfall event as indicated by the withe arrow: (**a**) Zoomed Gigapixel image of 14 May 2019; (**b**) Zoomed Gigapixel image of 15 May 2019 with the tilted rock block; (**c**) Zoomed Gigapixel image of 17 May 2019 after the failure.

From the analysis of the Gigapixel images, acquired every two hours, it was possible to identify both the debris movements on the cliff and at the base of the cliff. The detailed analysis of such images allowed us to also observe some precursory movements of the rock blocks, which later collapsed. An example is given in Figure 9, where it shown the evolution of the same rock block which moved two days before its collapse, progressively changing its dip angle.

### 4.2. Terrestrial ArcSAR Interferometry

The radar monitoring by using the HYDRA-G equipment was carried out with continuous acquisition for 72 h. Data were processed directly on-field by the Guardian software provided by IDS GeoRadar. Thanks to a rugged tablet it was possible to continuously check on-site the evolution of the monitoring parameters (e.g., displacement map and time series, reflectivity map, etc.) and the status of the system during all phases of the work.

**Figure 10:**
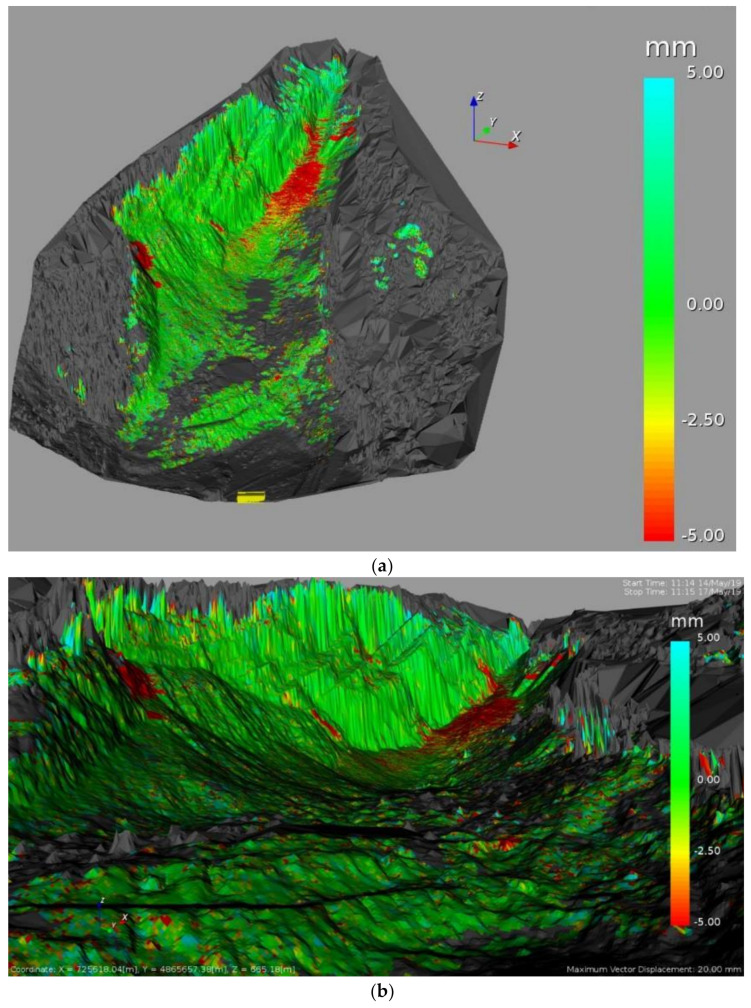
Line Of Sight (LOS) displacement map as result of 72 h monitoring of the Poggio Baldi landslide by HYDRA-G ArcSAR: (**a**) Landslide top view with the cumulative LOS displacement map overlaid on the 3D model of the slope; (**b**) The same LOS displacement map from the viewpoint of HYDRA-G.

**Figure 11:**
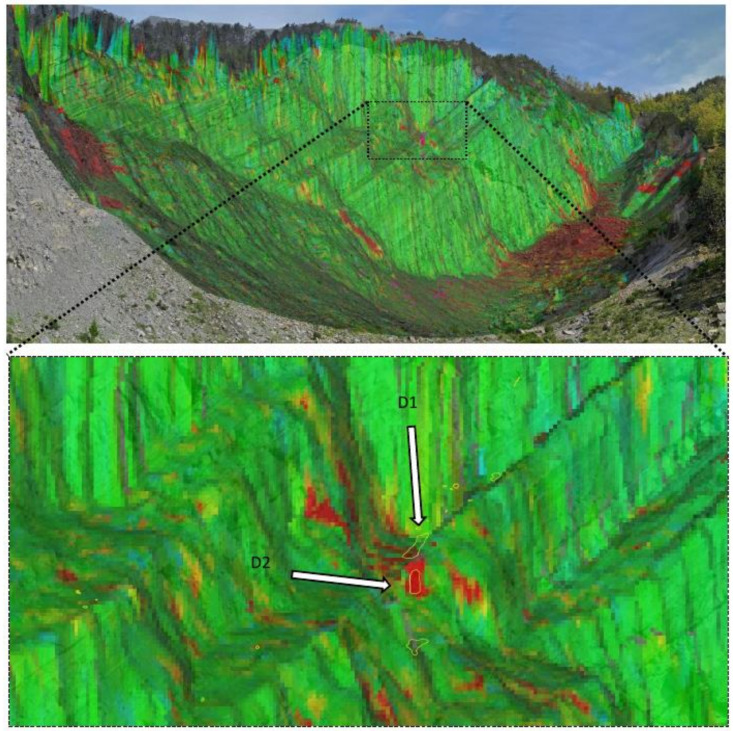
Central sector of the slope with correspondence between changes detected by radar measurements (green-red pixels) and GigaPan image analysis (yellow polygons); D1 and D2 polygons indicate areas that suddenly moved between 14 and 15 May 2019.

Following the data processing, all pixels containing the measurement information along the LOS (Radar-slope) were overlaid to a 3D model of the slope, generated by the instrument itself. Each pixel contains the cumulative displacement data of the 72 h of acquisition. As shown in Figure 10, the maximum cumulative displacement recorded during the 3-days monitoring by the ArcSAR instrument was about 5 mm (along LOS, approaching the sensor). The most active sector detected by the radar, displayed in red, was the debris talus located at the toe of the rock scarp with some portions bordering the scarp itself. In addition to the displacements recorded on the debris talus, a significant cumulative displacement of approximately 5 mm was identified in the central sector of the slope (Figure 11). According to the radar measurements, such areas (D1 and D2) suddenly moved between 14 and 15 May 2019.

### 4.3. Acoustic Measurements

**Figure 12:**
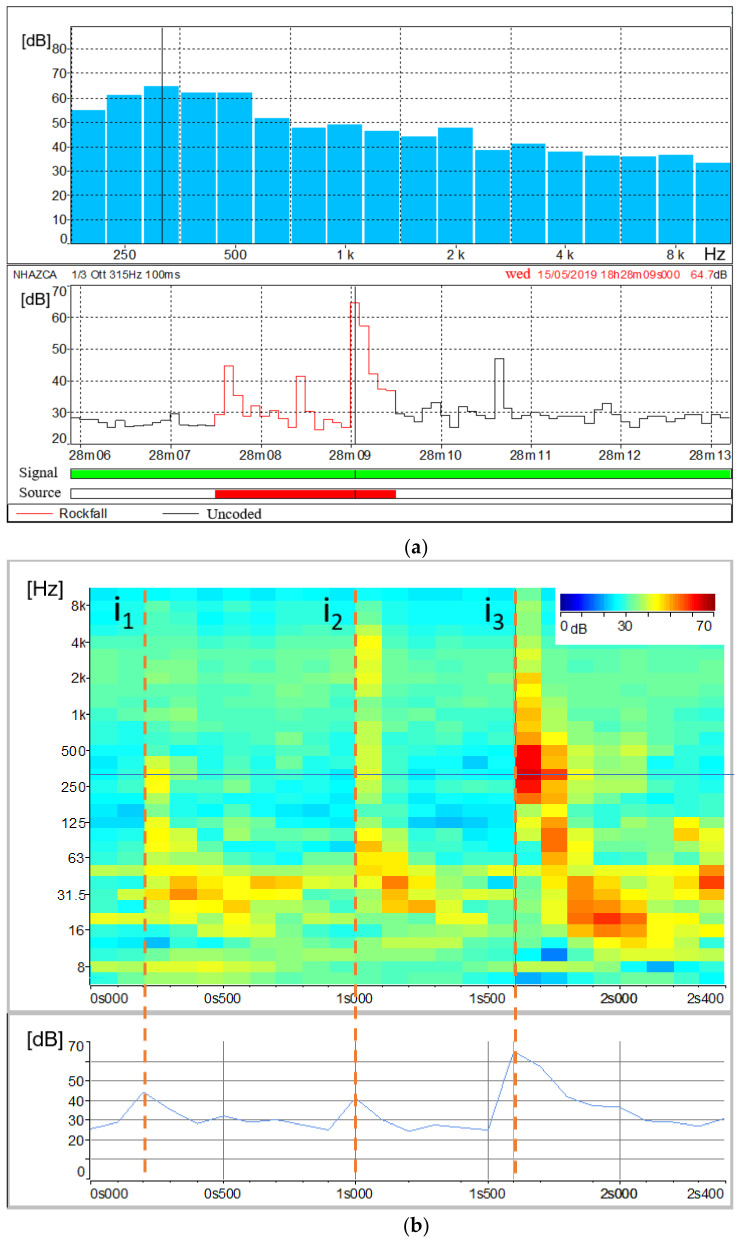
Overall representation of the acoustic signal emitted by the rockfall event on 15 May 2019 at 18:28 (UTC + 01:00) with its related impacts (i_1_, i_2_ and i_3_): (**a**) Spectral time history of the rockfall event with the classified soundtrack (lower plot) and frequencies histogram (upper plot); (**b**) Focus on the audio track classified as rockfall by means of the sonogram showing the amount of sound pressure levels expressed in dB as a function of time and signal frequency. The plot at the bottom shows the amount of sound pressure level over time at the frequency of 315 Hz. Temporal resolution is 0.1 s.

The collected acoustic signals allowed us to identify both the exact time of rockfall events and their signal characteristics in terms of sound pressure levels and spectral contents with a period of 100 ms. During the monitoring time span, three rockfall events were registered. The first one was artificially triggered by an operator by scaling a boulder in the middle part of the debris lying on the slope. This rock block felt for about ten metres before stopping at the bottom of the debris. By analysing the acoustic signal produced by the fallen rock block (Figure 12a), the final impact is clearly detectable (i.e., 15 May 2019, 18:28:09:000 UTC +01:00). Its noise peak reaches 64.7 dB at the frequency of 315 Hz (Figure 12a). The corresponding frequency histogram shows values above 60 dB with frequencies ranging between 250 and 500 Hz. Thanks to the sonogram, it was possible to investigate the amount of dBs for different frequencies and time steps (Figure 12b). In Figure 12b, three peaks at 315 Hz corresponding to different impacts occurred during the fall, can be observed (i_1_, i_2_, i_3_). Focusing on peak i_3_, its frequencies with the highest sound values (greater than 50 dB) range between 200 and 630 Hz, with relevant contributions up to 1 kHz (49 dB) and 2 kHz (47.4 dB). For all the three impacts, sound values above 40 dB are also reported for frequencies ranging from 16 Hz to 80 Hz. In detail, those values were recorded with a time delay of 0.1 to 0.6 s from the block impact time.

**Figure 13:**
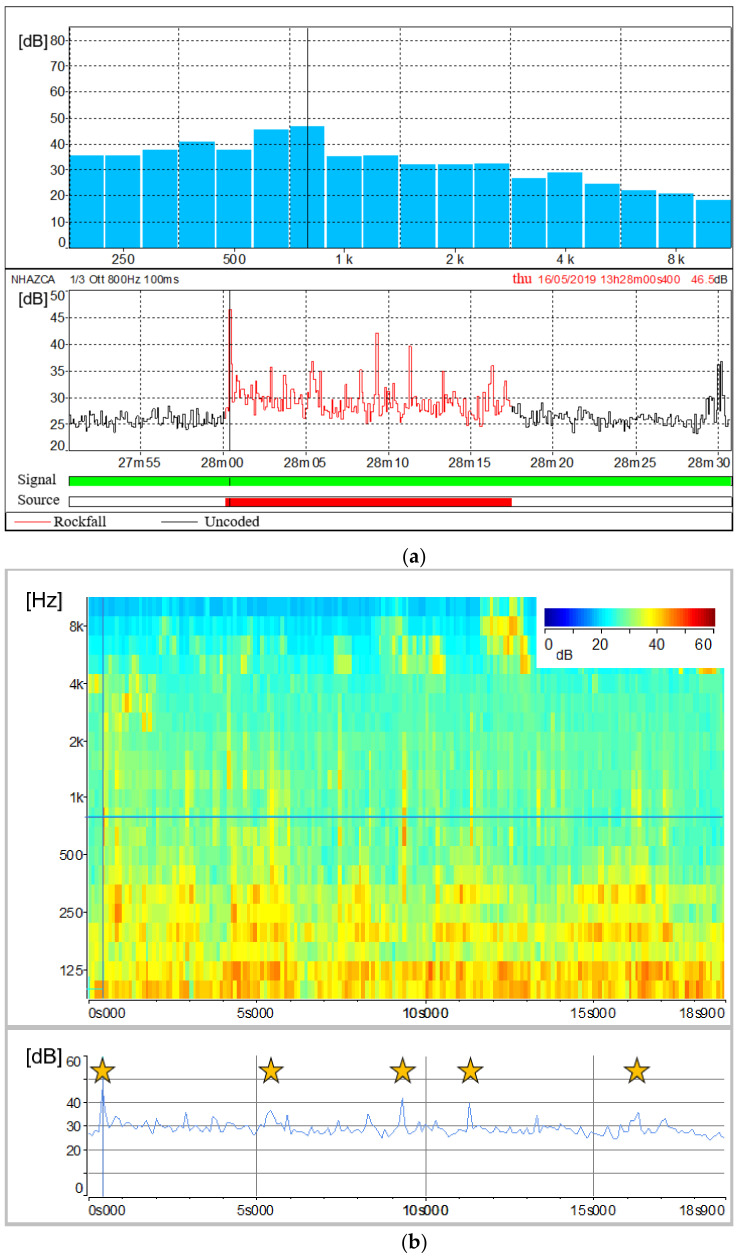
Overall representation of the acoustic signal emitted by the rockfall event on 16 May 2019 at 13:28 (UTC + 01:00): (**a**) Spectral time history of the rockfall event with the classified audio track (lower plot) and frequencies histogram (upper plot); (**b**) Focus on the audio track classified as rockfall by means of the sonogram showing the amount of sound pressure levels expressed in dB as a function of time and signal frequency. The plot at the bottom shows the amount of sound pressure levels over time at the frequency of 800 Hz. Yellow stars refer to different impacts recorded during the collapse. Temporal resolution is 0.1 s.

**Figure 14:**
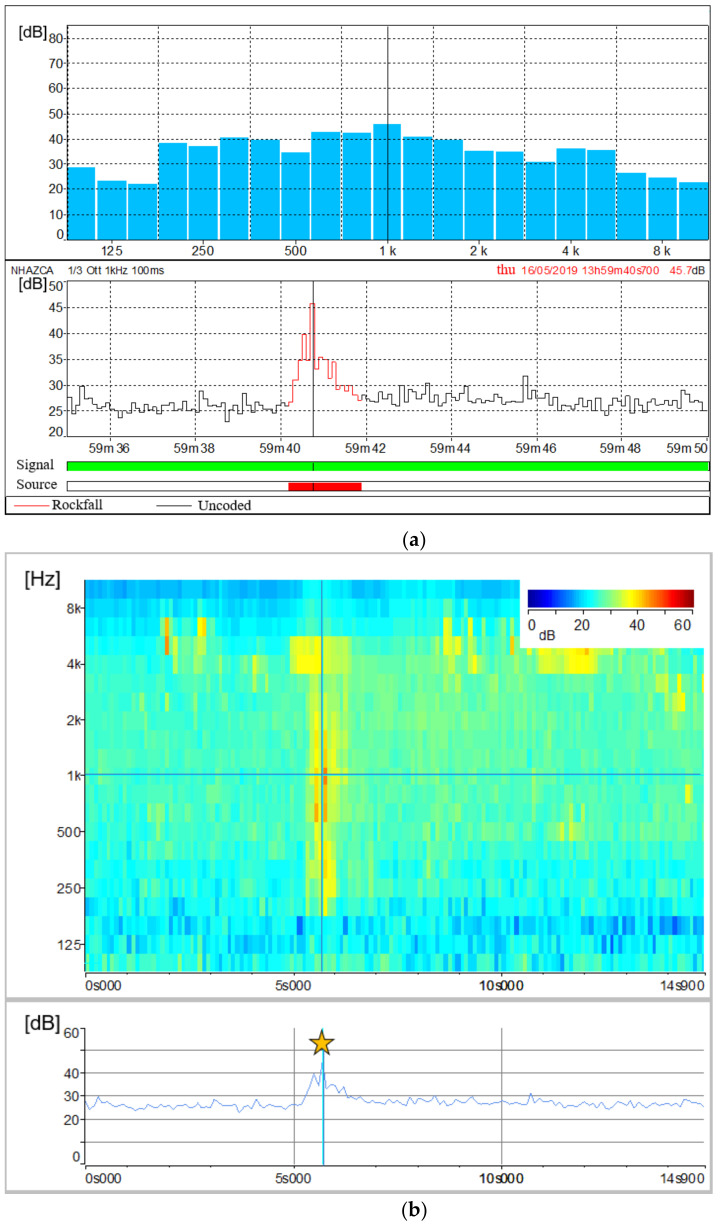
Overall representation of the acoustic signal emitted by the rockfall event on 16 May 2019 at 13:59 (UTC + 01:00): (**a**) Spectral time history of the rockfall event with the classified audio track (lower plot) and frequencies histogram (upper plot); (**b**) Focus on the audio track classified as rockfall by means of the sonogram showing the amount of sound pressure levels expressed in dB as a function of time and signal frequency. The plot at the bottom shows the amount of sound pressure levels over time at the frequency of 1 kHz. Yellow star refers to the time of the rock block detachment. Temporal resolution is 0.1 s.

**Figure 15:**
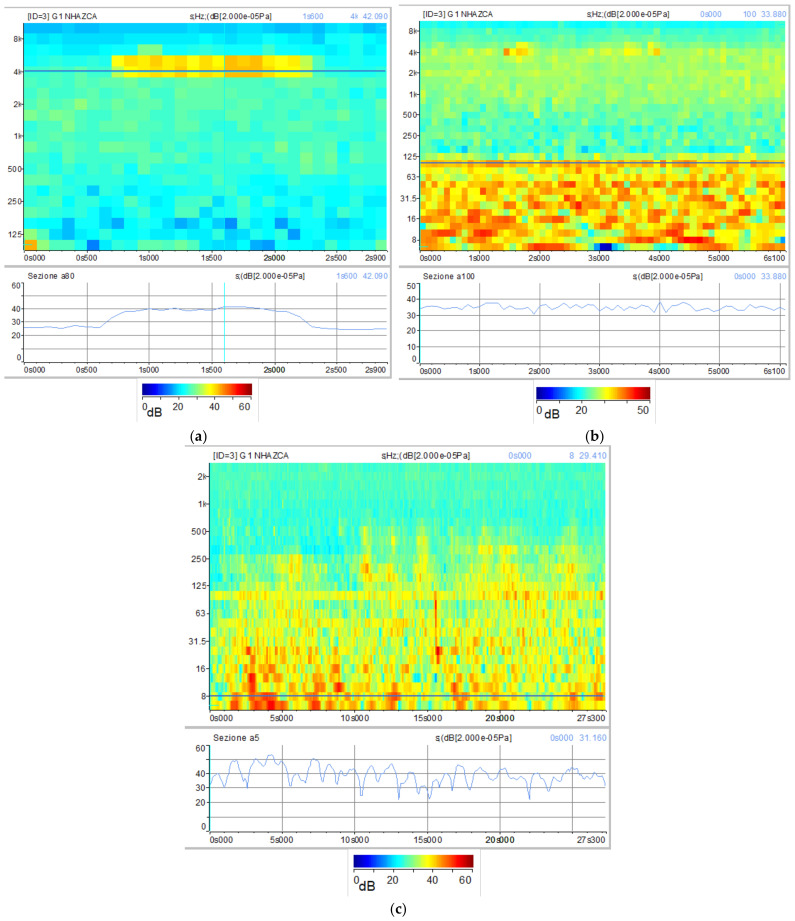
Sonograms referring to specific noise sources recorded during the acoustic monitoring campaign: (**a**) Birdsong; (**b**) Water runoff; (**c**) Aircraft noise. Line plots underlying each sonogram show the amount of sound level expressed in dBs over time at a fixed frequency (dark blue line).

Based on the results of the experimental rockfall, two different natural rockfalls detached from the rock cliff on 16 May 2019 at 13:28:00:400 and 13:59:40:700 (UTC + 01:00) were detected. Figure 13 and Figure 14 show the acoustic signal of the first and second rockfall, respectively. For the first one, significant noise from birds, water runoff and aircraft was recorded. However, among acoustic records, birdsong, water runoff and aircraft noise are depicted by frequencies of about 4–5 kHz, 8–100 Hz and 6.3–250 Hz respectively (Figure 15) allowing us to separate them from the rockfalls related spectra.

The 13:28 rockfall event was a long event with a total duration of 10.3 s including the detachment of the rock block from the cliff and the subsequent downfall marked by several impacts before stopping on the debris. Figure 13a shows in red the soundtrack section classified as rockfall and its frequencies histogram, which records the highest values of dBs between 630 Hz and 800 Hz (45.1 dB and 46.5 dB respectively), considering lower frequencies as introduced by the aircraft noise. The related sonogram confirms those values. At 800 Hz of frequency, different peaks relating to impacts occurred during the collapse are clearly evident (Figure 13b).

The 13:59 rockfall event was quite different from the previous one. It was recorded only the detachment phase without clear evidence related to its eventual impacts along the cliff and on the debris. Therefore, as shown by the classified soundtrack (Figure 14a), its total duration was about 2 s. This event produced the highest sound values between 630 Hz and 1.25 kHz, with its peak at 1 kHz (45.7 dB). The sonogram confirms the absence of subsequent impacts of the block and made it possible to distinguish bird noise (4–5 kHz) from the noise produced by the rockfall itself (Figure 14b).

## 5. Discussions

In the present study, the Gigapixel technique results in a high-performance tool, able to achieve images with a millimetric Ground Pixel Size (in the present case study up to 5 mm) from a sensing distance of several hundred meters (700 m). At the same time, it allows the capturing of RGB information useful in monitoring rockfalls and for the identification of lithological heterogeneities.

The 2D-CD allowed us to identify several rockfall events that occurred during the three-day monitoring campaign, precisely between 14–17 May 2019. Thanks to this technique, 84 failures along the rock cliff were identified and measured, many of which were attributed to rockfall phenomena even smaller than 1 m^2^ (down to 0.06 m^2^ at 500 m). The analysis of Figure 7 suggests that rockfalls mainly involved arenaceous mass and subordinately clayey strata. As noted by [47], they are clustered in the upper central portion of the rock cliff and accumulate at its toe. Furthermore, by means of several Gigapixel acquisitions within the same day, we were able to date rockfalls that occurred during the time span of two contiguous acquisitions (i.e., every two hours). Since only one rockfall event was detected through the 2D-CD performed with the pair of Gigapixel images acquired at 11:50 and 13:50 on 16 May 2019, it was possible to localise the source zone related to noise recorded at 13:28 by the 01dB DUO Smart Noise Monitor. Furthermore, knowing the exact Ground Pixel Size at that distance from the camera, we evaluated the areal dimensions of the collapsed rock mass in approximately 0.5 m^2^.

As the acquisition and analysis of Gigapixel images is time consuming, the synchronous use of the acoustic and optical sensor allowed a more efficient selection of Gigapixel images. These images were then processed for change detection purposes in order to localize the source area of the collapsed rock block.

As showed in Figure 9, major rockfalls might be preceded by precursory movements (i.e., pre-failure deformation) highlighting the proneness of certain rock slopes to have deforming areas and associated opening fractures that precede the main failure event [111,112]. Gigapixel techniques can also be combined with LiDAR products for further analysis aimed at three-dimensional characterisation and monitoring of rock slopes [113,114,115,116,117]. In [62,115] the Gigapixel images have been used to identify unweathered rock surfaces in order to map recent rockfall source zones. Another approach achieved by [113] consists of using such a technique to perform granulometric analysis of talus in rock slopes. In the case of PBL, we tested the application of 2D-CD to take the process one step further and present the use of Gigapixel images as a standalone tool to detect and date recent rockfall events as well as their geometrical features.

**Figure 16:**
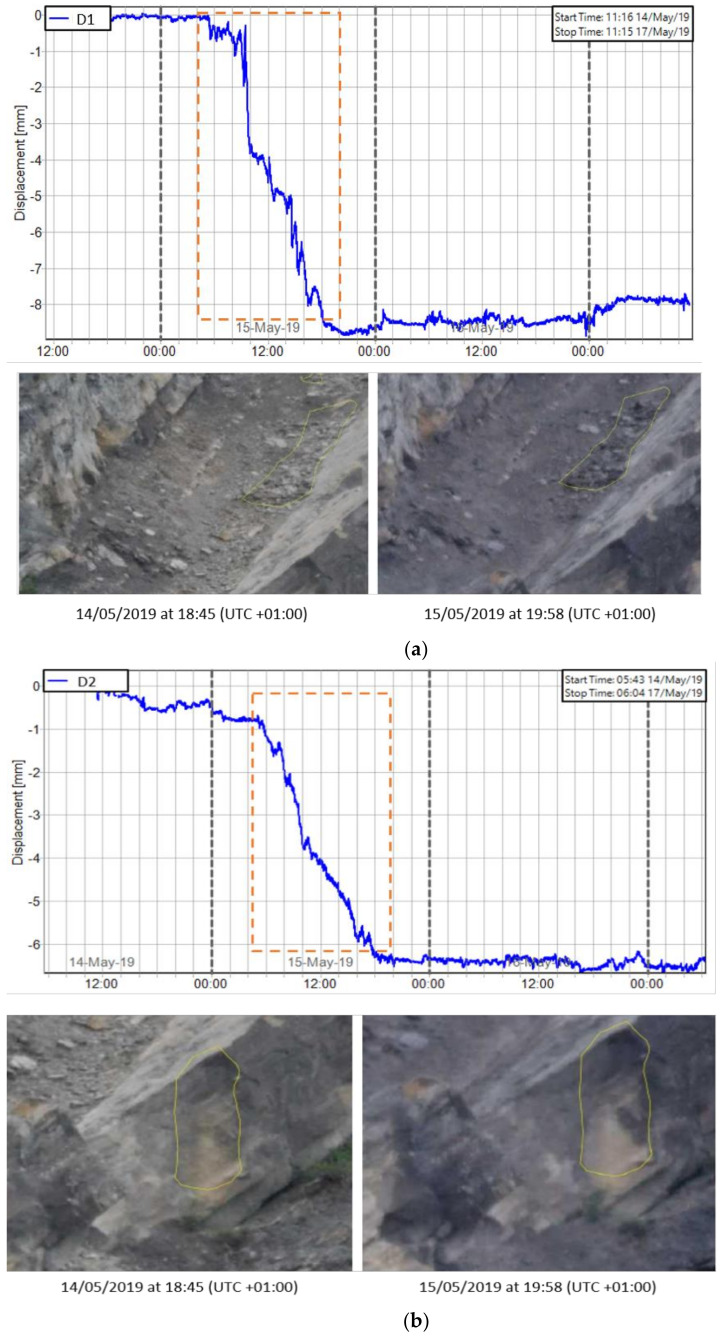
Comparison between radar measurements and Gigapixel analysis for D1 and D2 areas: (**a**) Displacement time series of D1 area measured by radar (above) and pre/post event images taken by GigaPan (below); (**b**) Displacement time series of D2 area measured by radar (above) and pre/post event images by GigaPan (below).

The use of Terrestrial ArcSAR Interferometry allowed us to perform a fully remote sensing monitoring of the Poggio Baldi landslide, without the need to place targets directly on the slope. Radar measurements were continuously carried out for 72 h (during both day and night) with acquisitions performed every 30 s. Thanks to its high temporal resolution and the integrated LiDAR, the radar was able to accurately date and locate the unstable processes occurred during the intensive monitoring campaign. In detail, between 14–15 May a sudden displacement of the D1 and D2 areas within the central sector of the slope was clearly detected. Such process was also confirmed by comparing the results from Gigapixel image analysis with the displacement time series recorded by the radar (Figure 16). In fact, D1 and D2 areas in Figure 16, were detected by the 2D-CD and interpreted as debris movements. However, radar system was not able to detect pre-failure deformations as observed with Gigapixel image analysis for the failure depicted in Figure 9. This could likely be due to the magnitude of deformation being above the level of detection (related to the wavelength of the radar system) or being shorter than the monitoring interval or due to monitoring geometry (e.g., no displacement along the LOS) or to the small size of the blocks, much smaller than the pixel size. Nevertheless, unlike the Gigapixel image analysis, radar data allowed to detect and quantify the displacement of debris talus at the toe of the scarp which reached a maximum displacement of 5 mm. Previous radar monitoring campaigns have been carried out at PBL using linear scanner TInSAR [56]. Thanks to the new monitoring campaign, through the use of HYDRA-G ArcSAR it was possible to focus the measurements within the unstable debris talus.

The complementary and combined use of sonograms relating to experimental and natural rockfalls together with sonograms relating to external noise sources, such as birdsongs, water runoff and aircraft noises, allowed us to study sound pressure levels of rockfall phenomena as well as their spectral contents. According to the results, we can state that features of acoustic signals vary with the volume and distance of the rockfalls from the microphone unit, as observed by [95]. This is also confirmed by [118], who affirms that landslide volumes are directly proportional to the spectral magnitude. Hence, a unique spectral signature of rock blocks falling from the Poggio Baldi landslide scarp is barely discernible. A further factor is the different sound attenuation level, which depends on the amount of atmosphere through which the sound waves travel. However, a narrow range of frequencies (200 Hz to 1000 Hz) can be designated as those most representative of rockfall signals. The assumption is validated by the experimental rockfall we triggered, which was both the least-affected by other noise sources and the closest to the microphone unit DMK01. In addition, according to [95], significant acoustic waves in the range of infrasonic frequencies (<20 Hz) up to 80 Hz were produced by the artificially triggered rockfall. However, as the other rockfalls recorded excessive noises in that frequency range, this spectral content can not be confirmed.

By comparing the three acoustic measurements we can also deduce different degrees in rockfalls fragmentation. Often, the detached mass breaks up on impact [107,119]. Rockfall fragmentation is the process by which the detached mass loses its integrity as it drops down a steep slope and shatters into smaller pieces [120]. Hence, the sonogram would show a series of minor randomly distributed peaks instead of a single well-defined impact.

As a result, for the artificially triggered rockfall (Figure 12), which did not exhibit fragmentation, the highest sound value is recorded at its third impact. Whereas we can assume a progressive and an instantaneous fragmentation respectively for the second and the third recorded rockfall (Figure 13 and Figure 14). For the second one, fragmentation is suggested by the gradual reduction in the acoustic waves power as the number of impacts increases; while for the third one, it is indicated by the absence of audible impacts during the collapse. In our opinion, these different behaviours in fragmentation are due to lithological composition (i.e., sandstone or marl) and/or various fracture intensities of the rock mass. However, further studies are needed to confirm this hypothesis.

## 6. Conclusions

The aim of this study was to evaluate the potential of innovative remote sensing rockfall monitoring systems and to characterize the very short-term gravitational processes ongoing at the Poggio Baldi landslide, with a specific focus in its upper part. For the first time, in-depth studies combining Terrestrial Interferometric ArcSAR, Gigapixel imagery and acoustic signals related to slope stability were carried out. The results of the monitoring campaign revealed the capabilities and limitations of each used tool: ArcSAR provided displacements of debris accumulated both at the toe of the scarp and on the arenaceous strata after the detachment. Gigapixel images have been analysed carrying out a 2D-CD in order to investigate the short-term evolution of the vertical rock cliff. This approach allowed us to detect and isolate several rockfalls events consisting of both sandstones and marls. Moreover, thanks to the short time interval of GigaPan acquisitions, pre-failure deformations prior to rock fall were observed. As well as ArcSAR data, Gigapixel image analysis was able to identify small deformations of the accumulated debris on the bulging strata surfaces that evolved into debris avalanches. Further, acoustic measurements were analysed in both acoustic decibel curves and time-frequency spectrum, revealing the ability of such sensors to detect failure occurrences and dominant frequencies affected by rockfall related signals. These unique properties could be used to automate the detection of future events.

Despite these capabilities, some open points in the assessment of PBL rockfall activity are still present. For instance, the obtained results could be combined with three-dimensional data such as point clouds, in order to achieve rockfalls frequency-volume relationships.

With reference to the integration of acoustic measurements and Gigapixel 2D-CD, we argued that the combination of these techniques should be intended as an integrated monitoring method aimed at improving the dating as well as the characterization—in terms of source zones, sizes, and lithology—of rockfall events. Following this approach, both high-resolution spatial and temporal distribution of rockfalls can be obtained.

In this perspective, future developments will be aimed at using both optical Gigapixel images and acoustic records for the automatic detection and distribution of rockfall events as well as the development of Digital Image Correlation (DIC) algorithms tailored for Gigapixel images, in order to detect and track slow deforming areas.

## Data Availability

Data is contained within the article or supplementary material.

## Supplementary Materials

The following are available online at https://www.mdpi.com/article/10.3390/s21082632/s1.
